# Decision-making preferences for intuition, deliberation, friends or crowds in independent and interdependent societies

**DOI:** 10.1098/rspb.2025.1355

**Published:** 2025-08-13

**Authors:** Igor Grossmann, Maksim Rudnev, Anna Dorfman, Mohammad Atari, Kelli Barr, Abdellatif Bencherifa, Wesley Buckwalter, Rockwell F. Clancy, German Cuji Dahua, Norberto Cuji Dahua, Yasuo Deguchi, Ancon Lopez Wilmer, Emanuele Fabiano, Badr Guennoun, Julia Halamová, Takaaki Hashimoto, Joshua Homan, Martin Kanovský, Kaori Karasawa, Hackjin Kim, Jordan Kiper, Minha Lee, Xiaofei Liu, Veli Mitova, Rukmini Nair, Ljiljana Pantovic, Brian Porter, Pablo Quintanilla, Josien Reijer, Pedro P. Romero, Yuri Sato, Purnima Singh, Salma Tber, Daniel Wilkenfeld, Lixia Yi, Stephen Stich, H. Clark Barrett, Edouard Machery

**Affiliations:** ^1^ University of Waterloo, Waterloo, Ontario, Canada; ^2^ University of Johannesburg, Johannesburg, South Africa; ^3^ Bar-Ilan University, Ramat Gan, Tel Aviv District, Israel; ^4^ University of Massachusetts Amherst, Amherst, MA, USA; ^5^ University of Pittsburgh, Pittsburgh, PA, USA; ^6^ Université Internationale de Rabat, Salé, Rabat-Sale-Zemmour-Zaer, Morocco; ^7^ George Mason University, Fairfax, VA, USA; ^8^ Virginia Tech, Blacksburg, VA, USA; ^9^ Kurintsa Community, Shiwiar Nation of Ecuador, Kurintsa, Pastaza, Ecuador; ^10^ Kyoto University, Kyoto, Kyoto Prefecture, Japan; ^11^ Universidad Antonio Ruiz de Montoya, Peru; ^12^ University of Coimbra, Coimbra, Coimbra District, Portugal; ^13^ Hassan II University, Casablanca, Grand Casablanca, Morocco; ^14^ Institute of Applied Psychology, Comenius University, Bratislava, Slovakia; ^15^ Toyo University, Bunkyo, Tokyo, Japan; ^16^ University of Kansas, Lawrence, KS, USA; ^17^ Institute of Social Anthropology, Comenius University, Bratislava, Slovakia; ^18^ University of Tokyo, Bunkyo, Tokyo, Japan; ^19^ Korea University, Seongbuk-gu, Seoul, Republic of Korea; ^20^ University of Alabama at Birmingham, Birmingham, AL, USA; ^21^ Seoul National University, Gwanak-gu, Seoul, Republic of Korea; ^22^ Wuhan University, Wuhan, Hubei, People’s Republic of China; ^23^ Indian Institute of Technology, Delhi, Delhi, India; ^24^ University of Belgrade, Belgrade, Serbia; ^25^ Pontificia Universidad Catolica del Peru, Lima District, Lima Region, Peru; ^26^ Universidad San Francisco de Quito, Quito, Pichincha, Ecuador; ^27^ Ochanomizu University, Bunkyo, Tokyo, Japan; ^28^ Indian Institute of Technology Delhi, New Delhi, India; ^29^ Rutgers The State University of New Jersey, New Brunswick, NJ, USA; ^30^ University of California at Los Angeles, Los Angeles, CA, USA

**Keywords:** decision-making, folk theories, culture, descriptive norms, social norms, social orientation, wisdom

## Abstract

When multiple ways of deciding are laid out side-by-side, which does one favour? We conducted experiments in 12 countries (*n* = 3517 individuals; 13 languages; two Indigenous communities), with adults choosing among four decision strategies—personal intuition, private deliberation, friends’ advice or crowd wisdom—when working through six everyday dilemmas. In every society, self-reliant decisions (intuition or deliberation) were most commonly preferred and considered the wisest. Expectations for fellow citizens, however, were mixed: advice from friends was expected about as often as self-reliant routes. The self-reliance tilt was strongest in cultures and individuals high in independent self-construal and need for cognition, and weakest where interdependence and self-transcendent reflection were salient. The same patterns emerged when examining ratings of each strategy’s utility and oral protocols with Indigenous groups. Self-reliance appears the modal preference across cultures, but its strength is predictably tempered when cultures, and individuals within them, construe the self in relational rather than autonomous terms.

## 1. Introduction

When consequential choices loom (e.g. investing family savings, changing careers, deciding which crop to plant), people can rely on a wide range of approaches. They may tune in to gut feelings formed by past experience, reason methodically on their own, recount the dilemma to trusted friends and adopt their counsel, or even seek crowd wisdom by posting the problem to a broad, anonymous audience and following the modal reply. The first pair are self-reliant strategies that draw solely on one’s experiences or deliberations; the second pair are advice-oriented strategies that foreground other minds. Here we ask, in light of cultural evolutionary theories predicting openness to outside counsel [1,2] and the opposing advice-discounting evidence drawn mostly from Western, educated, industrialized, rich and democratic (WEIRD) laboratories [3], which of these four approaches people around the world *prefer* when all are offered, and how that preference shifts with culture and self-construal.

Decision scientists have shown that seeking and incorporating advice from others can enhance individual reasoning and decision-making [4–8]. Anthropologists and evolutionary biologists, in turn, have argued that features of social learning such as prestige bias serve social coordination and facilitate human cultural evolution, allowing humans to adapt and thrive [1,2,9–11]. If effectiveness alone dictated preference, advice-oriented strategies should be attractive.

Yet scores of laboratory studies reveal the opposite pattern. After hearing an adviser’s estimate, participants substantially under-weight it, revising their own answer far less than normative models recommend [4]. When advice seeking is optional and costless, many decision makers request it sparingly or disregard it once received. These findings constitute the advice-discounting bias: people act (or expect to act) as though their private information is sufficient. The puzzle is why a species that prospers by sharing knowledge so often voices a preference for deciding alone [11].

One can draw on two viable explanations. On the one hand, advice-discounting can be an anomaly dominant in societies cultivating independent self-construals and celebrating personal agency, making self-reliance the default and advice-oriented decisions a sign of weakness [12,13]. By this logic, societies that foreground interdependent self-construal should lean towards viewing interpersonal considerations as central to their choice [14,15]. On the other hand, advice-discounting may spring from broad motivational and cognitive regularities—autonomy needs make self-reliant acts inherently rewarding [16]; naive realism leads people to treat their own view as uniquely objective [17]; and ego-centric accessibility renders private evidence more vivid than an adviser’s [18]. In this view, self-reliant strategies should remain the modal preference across cultures, with cultural and personality factors modulating—but rarely overturning—the tilt toward others’ advice.

Existing evidence is inconclusive. Some cross-national surveys suggest that managers in certain countries (e.g. Japan, Ghana) may be more inclined to seek input from colleagues than in more individualistic settings like the US, although this pattern is not consistently found in interdependent cultures [19]. Other surveys point the opposite way: East Asians sometimes avoid burdening others with requests for help [20] and show lower interpersonal trust, which can suppress advice use [21]. Nearly all of this work is limited to two-country contrasts or student samples, leaving much of the world unexplored [22–24] and mixed findings unresolved.

The present study provides the first large-scale adjudication of these accounts. Across 20 samples, we surveyed 3517 adults speaking 13 languages in 12 countries—spanning the Global North (higher-income, industrialized) and Global South (lower-/middle-income, post-colonial) and including two small-scale Indigenous communities. For six everyday dilemmas (co-designed with local collaborators), participants (i) selected the single strategy they *preferred*, (ii) rated the wisdom of each strategy, (iii) predicted what ‘most people in your culture’ would do and (iv) provided ratings of expected utility of each strategy on a rating scale (i.e. the expectation to feel good about the choice [25]). They also completed validated measures of independent vs interdependent self-construal, need for cognition and reflective style.

Following dominant cultural psychological frameworks [e.g. 9,13,26,27], if advice-discounting is culturally bounded*,* self-reliant choices should weaken—or reverse—in interdependent cultures and among individuals who endorse interdependence. Conversely, if it is a human regularity, self-reliant strategies should remain the modal preference everywhere, with self-construal and cognitive style modulating its strength but not its direction.

Our study draws on a markedly diverse sample. We contrast explicit preferences for intuition, deliberation, friends’ advice and crowd wisdom, then situate those preferences within participants’ folk theories of wise decision-making. Together, these steps provide the first large-scale test of whether advice-discounting is an anomaly or instead a broadly recurrent stance whose magnitude is calibrated—but not overturned—by culture.

### (a) Study overview

To achieve our research goals, we conducted a three-stage study: exploring general tendencies, focusing on under-reached populations and testing the results using modified stimuli. In a repeated-measures design in Stage 1, participants confronted six dilemmas: some required choosing between two similarly attractive, incommensurable options, while others pitted self-interest against the interests of others. The scenarios were developed in consultation with the project’s ethnographers and anthropologists. For each scenario, participants indicated their preference for advice-oriented strategies (friends’ advice and wisdom of crowds) or self-reliant strategies (self-focused intuition and deliberation) and indicated how they would feel when choosing each strategy on a rating scale (allowing to test for relative preference of particular strategies). They further completed measures of self-construal, need for cognition, reflective styles and solution-seeking tendencies. To examine if the present results extend beyond the industrialized communities, in Stage 2, we tested participants from two rural Indigenous communities in South America, using a shortened version of the initial protocol in the form of verbal interviews. In the confirmatory Stage 3, we followed the same procedure on a subset of four dilemmas, with a minor modification: whereas Stage 1 decision strategies included a clause of omission of other strategies to highlight the single source of information (e.g. for ‘deliberation’: decision based on thoughts and reasoning, ignoring gut feelings or what others say), in Stage 3, we used a more naturalistic prompt without the omission clause (e.g. ‘deliberation’: decision based on thoughts and reasoning). Finally, in a supplementary study on an ethnically heterogeneous North American sample, we simultaneously tested preferences across ethnic groups from the same country, probing responses across two modalities—as a forced choice method and via rating scales of internal and external strategies as wise, rational and culturally acceptable.

## 2. Methods

### (a) Participants

Participants were recruited through the Geography of Philosophy Project, a consortium of 12 collaborating research teams (table 1). Each team followed its own locally appropriate recruitment procedures, but all respondents completed the same standardized interview battery. Most sites sampled both university students and non-university community members. Stage 1 respondents were overwhelmingly native-born residents of the country in which they were tested (electronic supplementary material, table S1). Stage 2 focused on Shiwiar and Shipibo villages in Ecuador and Peru—Indigenous Amazonian horticultural societies that combine village-level collective decision-making with household economic autonomy^1^ [28]. Samples differed widely in demographic and socio-economic composition and remuneration (electronic supplementary material, tables S1–S2). Overall, 52% of participants identified as female, and education levels ranged from primary school to graduate degrees. All sites met or exceeded the pre-registered sample-size targets for the within-subject design. US subsamples (University of Pittsburgh students and online crowdworkers) and Indian subsamples (Tamil- and Meitei-speaking adults in Delhi) were aggregated to satisfy those targets (see electronic supplementary material, Methods, for power considerations).

**Table 1.** Descriptives in each site. Study language used is in parentheses.

stage	site	*M*_age_	*SD*_age_	female (%)
1	Canada (English; *n* = 152)	20.27	3.24	84.0
	China (Mandarin; *n* = 334)	30.34	7.51	56.3
	Ecuador (Spanish; *n* = 143)	21.06	2.38	58.7
	Germany (German; *n* = 147)	39.74	12.04	37.6
	India (Hindi; *n* = 114)	26.12	7.41	46.8
	India (Tamil/Meitei; *n* = 152)	30.87	11.13	65.4
	Japan (Japanese; *n* = 318)	29.66	10.91	48.0
	Morocco (Arabic; *n* = 99)	22.11	7.80	57.7
	South Africa (English; *n* = 270)	25.45	8.44	65.0
	South Korea (Korean; *n* = 179)	23.07	3.65	62.0
	Slovakia (Slovak; *n* = 249)	29.26	12.34	28.9
	USA (English; *n* = 219)	36.33	12.72	48.2
2	Shipibo, Peru (Shipibo; *n* = 73)	36.30	12.15	56.2
	Shiwiar, Ecuador (Shiwiar; *n* = 45)	38.93	15.03	55.6
3	Canada (English; *n* = 140)	31.51	10.83	47.1
	China (Mandarin; *n* = 401)	29.09	7.83	52.6
	India (Hindi; *n* = 56)	30.13	5.18	71.4
	Morocco (Arabic; *n* = 164)	32.60	10.80	43.2
	Peru (Spanish; *n* = 198)	22.76	3.85	65.1
	South Africa (Zulu; (*n* = 111)	30.77	8.37	43.8

### (b) Procedure and materials

Pre-registered analysis plan, deviations and exclusion criteria are in the electronic supplementary material. Materials were translated with back-translation and expert consensus (see electronic supplementary material for further details).

#### (i) Scenarios

In Stage 1, participants were presented with six decision-making scenarios that varied in complexity and interpersonal stakes. To ensure cultural relevance, all scenarios were selected based on the inputs of the anthropologists, philosophers and linguists on the research team; participants in each sample reported moderate-to-high personal relevance of each scenario, as indexed by feeling of closeness to the protagonist (electronic supplementary material, figure S1). The scenarios included agricultural assistance, financial investment, educational choices, travel planning, neighbourly assistance and academic support. The diversity in scenarios afforded exploring decision-making across different contexts and trade-offs, including time, resources and opportunities. The following scenarios, used in all sites, illustrate the types of decisions participants considered:

— *Thanks to the sale of land owned by his grandfather, José has obtained an abundant amount of money that he wants to use for his family. The amount at his disposal is enough to buy an orchard or a herd of animals for his family. José knows that each option could be very useful to improve the life of his family and a secure job for his sons, but he has to pick only one*. [Scenario involving two attractive options.]
— *Carlos, a young man, asks Marcos, his neighbour, if he can help him prepare his field to be able to plant before the rainy season. Marcos has already started working in his field a few weeks ago and knows that if he helps his neighbour Carlos he will not be able to finish all the remaining work on time, and his harvest will not be as abundant as expected*. [Scenario involving self-interest and others’ interests.]

Half of the scenarios involved a choice between two *attractive options* such as deciding between buying an orchard or a herd of animals (in José’s scenario above), choosing a university to pursue one’s degree, or selecting a travel destination (see verbatim materials in the electronic supplementary material). As in José’s example above, these scenarios were designed to examine decision-making when each option appears beneficial to the decision-maker but distinct in their outcomes, and the options are incommensurable. The other half of the scenarios involved a dilemma between *self-interest and others’ interests* with ambiguous outcomes, such as deciding whether to help a neighbour at the expense of personal work deadlines (in Marcos’s scenario above) or balancing personal study time with assisting a struggling friend. Critically, most scenarios involved decisions that had significant consequences for the decision-maker and others. For example, Marcos’s decision to help his neighbour could significantly impact both parties' agricultural productivity, whereas an educational choice—which university to attend—could have long-term career implications.

We expected that study completion would be slower with some populations not used to surveys. For such pragmatic reasons, some samples (e.g. working-class Meitei community in New Delhi, time-constrained South African college students) responded to a subset of the scenarios (see electronic supplementary material, table S3 for demographic-specific scenario assignments). When we tested participants from two small-scale communities in South America in Stage 2, we relied on a shortened version of the Stage 1 protocol in the form of verbal interviews. Specifically, participants were presented with a subset of two stories designed to match the socio-ecological context of participants from these communities (see verbatim in the electronic supplementary material). In Stage 3, we selected four scenarios from the original six used in Stage 1 (see electronic supplementary material, table S3 for selected scenarios).

#### (ii) Procedure

Participants read each scenario and then were told that there are ‘several ways this person could handle the situation’, then prompted to evaluate four distinct strategies, all presented on the same screen to highlight the contrast between them. Two strategies chiefly relied on one’s own inner mental processes (*gut feeling* or *deliberation*). The other two strategies involved advice from other people (*friends* or ‘*wisdom of crowds*’). Description of each strategy started with distinct information gathering processes, before highlighting that the decision will be made specifically based on this information source (see table 2).

**Table 2.** Overview of decision-making strategies with process steps presented to participants.

steps	personal intuition	personal deliberation	friends’ advice	wisdom of crowds
collecting information	X spends some time tuning in to her feelings and emotions about the situation *on her own*. X attends to her gut feeling about the situation, her personal experiences and her emotions.	X spends some time reasoning and reflecting on the situation *on her own*. X reasons about the situation, her personal experiences and her thoughts.	X spends some time *getting the opinion of her other friends* about the situation. X describes the situation to her friends. Her friends listen and offer advice.	X spends some time *getting the opinions of a number of strangers* about the situation. X posts an anonymous question online describing the situation. People read about the situation and offer advice.
making decision	X then makes her decision based on her gut feeling,	X then makes her decision based on her thoughts and reasoning,	X then makes her decision based on her friends’ advice,	X then makes her decision based on the strangers’ most common advice,
exclusive choice	Even if it does not align with her thoughts and reasoning, or with what others may think about the situation.	Even if it does not align with her gut feeling, or with what others may think about the situation.	Even if it does not align with her thoughts and reasoning, or with her gut feeling about the situation.	Even if it does not align with her thoughts and reasoning, or with her gut feeling about the situation.

Note. X = local names typical to a given country for each role and location (see electronic supplementary material, table S2). Stage 2 participants were presented with a simplified 'wisdom of crowds' option involving an anonymous public board rather than the internet (see electronic supplementary materials for verbatim text). Following the standard definition of wisdom of crowds, in Stages 1−2 advice was presented as a variable to simulate heterogeneity of suggestions—i.e. the advice these people gave about the situation varied. In Stage 3, we omitted this sentence to ensure earlier findings were not due to participants favouring more certain options. In Stages 1-2, the 'making decision' sentence continued after a comma into the line presenting the 'exclusive choice'. In Stage 3, we omitted the 'exclusive choice' text to allow a more naturalistic blending of approaches; thus, the 'making decision' sentence ended with a full point.

In Stages 1−2, we concluded with a sentence underscoring that the final decision is based only on that one source—irrespective of what other thoughts or advice might suggest (see bottom ‘exclusive choice’ row in table 2; see verbatim text in the ‘Choice Options’ section of the electronic supplementary material). As pre-registered, Stage 3 removed these exclusionary statements to allow more naturalistic blending of information sources, while still focusing on the dominant information source for their decision-making.

In Stages 1 and 3, strategies were presented either online on the Qualtrics platform, or via paper and pencil (Slovakia). In Stage 2, team members interviewed participants from Indigenous societies, with responses recorded by the interviewer (see electronic supplementary material, table S2 and the general script for administration for paper and pencil/interview sites in the electronic supplementary material). To control for order effects, scenario order was randomized (online)/counter-balanced (paper and pencil).

## 3. Measures

For each scenario, participants chose which strategy reflects their personal preference, the strategy they considered to be wisest and the one most people from their cultural background would use, followed by scale-based rating of each strategy’s anticipated subjective utility, and a set of individual difference measures.

### (a) Personal preference

To examine personal preferences, participants selected one of the four advice- and non-advice-oriented strategies, when responding to the question: ‘Which strategy would you use?’

### (b) Perceived wisdom

We asked participants to provide the evaluative judgment of the perceived wisdom to examine whether people view their own personal choice preferences as appropriate, responding to the prompt: ‘Please choose the strategy that you consider the wisest’. Building on self-enhancement theory [29,30], we expected personal preferences to go hand-in-hand with strategies they would deem wise.

### (c) Descriptive norm

In addition, we probed cultural differences in false consensus of the advice-oriented decision preferences (i.e. expectations that most others from their culture would use the same strategy). To this end, we asked ‘Which strategy are most people from *your cultural background* likely to use?’ to subsequently evaluate differences between personal preferences and descriptive norms across samples varying in self-construal.

### (d) Anticipated subjective utility

Subsequently, participants assessed the expected utility of each strategy on a 5-point scale. The rating-scale approach allowed for assessment of the degree to which participants viewed each strategy as preferred, testing for similarity and dominance in decision preferences. Participants responded to the following question: ‘To what extent would choosing each strategy make you feel *good* about yourself?’ (1 = *not at all*; 2 = *slightly*; 3 = *moderately*; 4 = *very*; 5 = *extremely*).

### (e) Independence and interdependence

Participants completed a 22-item scale to assess their self-construal [31]. The scale includes statements measuring independent orientation (e.g. ‘My personal attributes are what make me who I am’) and interdependent orientation (e.g. ‘My relationships with others are a very important part of who I am’).^2^ See psychometrics evaluations of this and other individual difference instruments, including cross-cultural alignment procedure [32] for measurement equivalence tests in the supplementary results (see electronic supplementary material, tables S10–S13).

### (f) Need for cognition

The need for cognition refers to people’s tendency to engage in and enjoy thinking [33]. To assess individual differences in motivation to engage in effortful thinking, participants completed the 18-item need for cognition scale (e.g. ‘I would rather do something that requires little thought than something that is sure to challenge my thinking abilities’).

### (g) Self-transcendent, meta-cognitive and conflict-resolution style

Finally, to probe the role of reflective and social cognitive tendencies, we asked participants to complete the Situated Wise reasoning Scale (SWiS) [34,35]. Following an event-reconstruction protocol, participants recalled and reconstructed a recent interpersonal disagreement with another person. Subsequently, they reported to what extent they reflected on the events from the perspective of an outsider (*self-transcendence*; 4 items; example item: ‘Tried to see the conflict from the point of view of an uninvolved person’), recognized diverse viewpoints and acknowledged limits of their knowledge (*perspectival meta-cognition*; 8 items; example item: ‘I looked for alternative explanations before forming my opinion’) and considered the possible solutions to the problem (*solution-seeking*; 9 items; example item: ‘I looked for different solutions for this situation’). The three-factor model is based on the preliminary cross-cultural analyses suggesting that it reflects the most robust solution across most of the samples [36]. For pragmatic reasons, we employed a short version of the instrument in Stage 2 (*self-transcendence*: 2 items; *meta-cognition*: 4 items; *solution-seeking*: 4 items; see electronic supplementary material for verbatim items). At the end, participants provided basic demographic information (see table 1).

To reduce the cognitive load and ensure consistency across scales, and thereby increase reliability, for the individual difference measures, we standardized response options to a 5-point scale (1 = s*trongly disagree,* 2 = s*omewhat disagree*, 3 = *neither agree nor disagree*, 4 = s*omewhat agree*, 5 = s*trongly agree*).

### (h) Analytic strategy

We contrasted advice-oriented strategies (friends’ advice and wisdom of crowds) with self-reliant strategies (gut feeling and personal deliberation) using generalized linear mixed-effects logistic regressions. Responses were nested within scenarios and participants, and participants within samples; fixed effects captured overall trends, and random intercepts allowed for scenario and cultural clustering. Analogous mixed-effects models were run for anticipated-utility ratings and other outcome measures.

Preregistered predictions about cultural variation were tested in a mega-analysis that pooled data from all three project stages and added sample- and person-level self-construal predictors. Finally, a separate model evaluated whether advice preference differed between personal dilemmas and social dilemmas. Model specifications and details are provided in the electronic supplementary material.

## 4. Results

### (a) Advice-discounting across cultures: exploratory analyses

**Figure 1.**
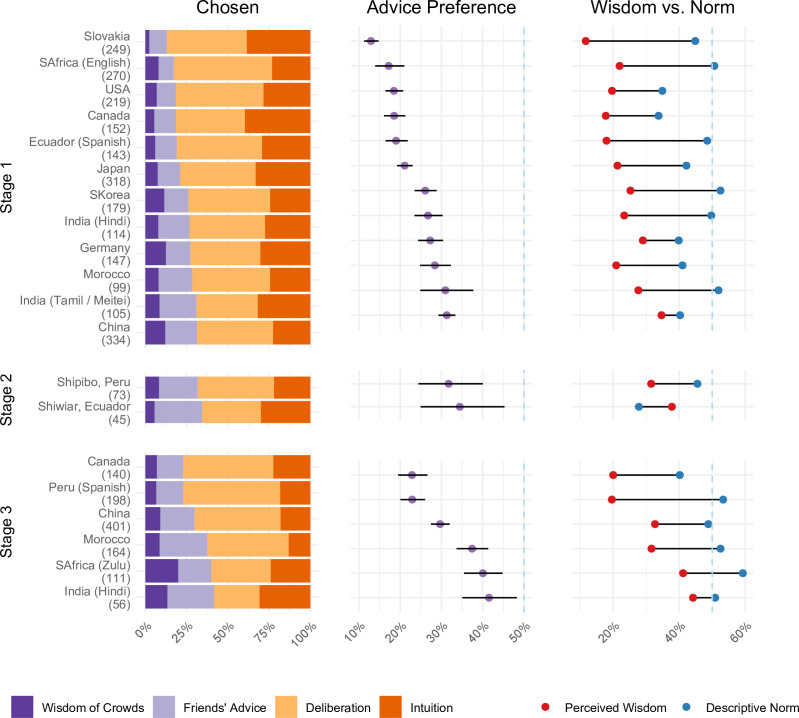
Decision-strategy preferences by study site. Left: observed choice proportions for advice-oriented strategies—friend advice, crowd wisdom (purple)—versus self-reliant strategies—deliberation, intuition (orange); countries ordered by advice dominance (*n*). Middle: model likelihood of choosing advice (95% *CI*); all <50%. Right: advice preference for *wisest* choice and descriptive norm.

When asked about their personal preference for a decision strategy, Stage 1 participants chose personal deliberation most often, ranging from 37% among Tamils and Meitei in India to 60% among English-speaking South Africans (see left part of figure 1). Intuition was consistently the second most chosen strategy, ranging from 23% among the participants from China to 40% among the participants from Canada (top left panel of figure 1).^3^ In all countries, the two advice-oriented strategies were visibly less popular: preference for friends’ advice ranged between 9% in South Africa and 22% among Tamils and Meitei, whereas wisdom of crowds advice was preferred only between 2% in Slovakia to 12% in Germany. Formal comparisons between advice-oriented and self-reliant strategies indicated that the latter were dominant, with prevalences ranging from 69% in China to 87% in Slovakia, *B* = 83%, 95% CI [74%, 89%], *z* = 5.85, *p* < 0.001, *OR* = 4.92. Results were almost identical when estimating effects for people who self-identified as being native to respective countries, *B* = 84%, 95% CI [76%, 90%], *z* = 6.06, *p* < 0.001, *OR* = 5.32.

We replicated these results in two Indigenous South American communities (Stage 2 in figure 1), with participants favouring self-reliant strategies, *B* = 72%, 95% CI [62%, 80%], *z* = 3.94, *p* < 0.001, *OR* = 2.62. Moreover, we observed very similar results in the confirmatory Stage 3, where we did not frame strategies as mutually exclusive. As figure 1 shows, personal deliberation was the preferred strategy everywhere except in India (where participants favoured intuition); overall participants favoured self-reliant over advice-oriented strategies, *B* = 72%, 95% CI [58%, 82%], *z* = 2.99, *p* = 0.003, *OR* = 2.52.

#### (i) Anticipated utility of decision strategies

**Figure 2.**
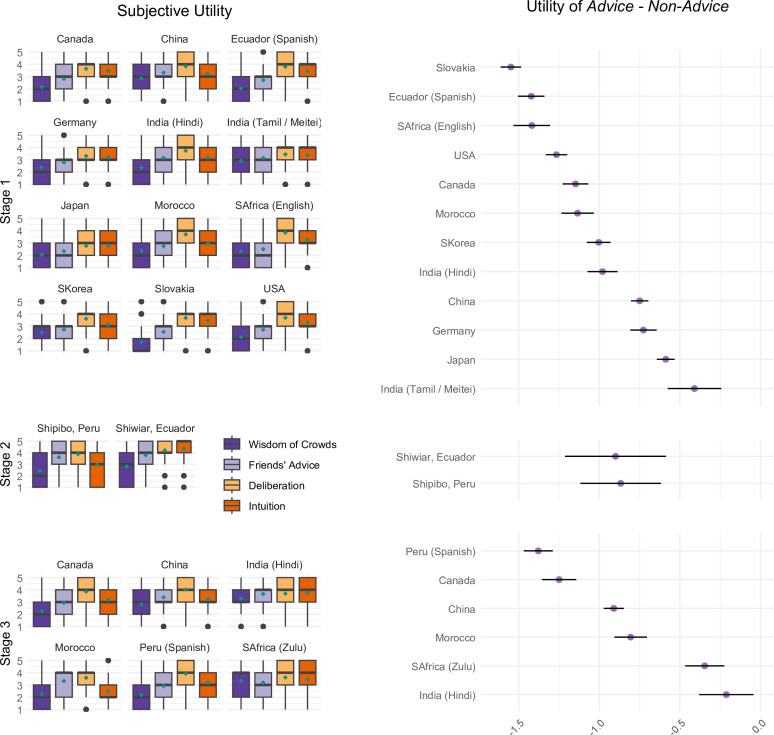
Cultural variation and systematicity in anticipated self-evaluative utility—sense of feeling good about oneself (1 = not at all … 5 = extremely). Left: boxplots by strategy—median line, IQR box, 1.5 IQR whiskers, outliers—plus model means (teal diamonds). Right: model contrast advice—self-reliant (95% *CI*); all *CI* boundaries < 0, showing higher ‘feel-good’ ratings for self-reliant over advice-oriented strategies.

To ensure these results were not a methodological artifact of forced-choice responses, next we examined rating-scale based markers of anticipated subjective utility of each strategy (figure 2; see further evidence from rating-scale responses in electronic supplementary material, figure S6). In all samples, except the Shipibo, participants were significantly more likely to anticipate feeling worse about choosing advice-oriented strategies, and the cultural differences closely mirrored the forced‑choice results shown in figure 1, Stage 1: *B* = −0.88, 95% CI [−1.14, −0.62], *t*(13.41) = 6.57, *p* < 0.001; Stage 2: *B* = −0.68, 95% CI [−4.27, 2.91], *t*(1.00) = 2.45, *p* = 0.248; Stage 3: *B* = −0.54, 95% CI [−0.89, −0.19], *t*(7.86) = 3.00, *p* = 0.003.

#### (ii) Perceived wisdom and descriptive norm

As figure 1 shows, similar results emerged when we asked participants about the perceived wisdom, with personal deliberation perceived as the wisest strategy in most samples (36–61%), followed by intuition (15–36%), with fewer people selecting friends’ advice (9–19%) or wisdom of crowds (3–16%). However, results looked different when examining descriptive norms. Though wisdom of crowds was perceived as the least used strategy by the majority in one’s culture (ranging from 6% among participants from Canada to 17% among participants from Germany), expectations for other members of one’s culture to seek advice from a friend were not different from intuition and personal deliberation. In fact, in five countries—South Africa, Ecuador, South Korea and India—seeking advice from friends was viewed as the strategy others would most commonly choose. Notably, even among those participants who indicated that others in their culture would seek advice, the majority personally preferred self-reliant strategies, *B* = 70%, 95% CI [58%, 79%], *z* = 3.22, *p* = 0.001, *OR* = 2.32—culture-specific analyses revealed a consistent trend for most sites (from 63% in South Korea to 87% in South Africa), with the exception of Germany (53%) and China (42%).

We replicated these results in two Indigenous South American communities (Stage 2), and in a separate study where we did not present strategies as mutually exclusive (confirmatory Stage 3): Participants viewed self-reliant strategies as wiser than advice-oriented strategies, Stage 2: *B* = 71%, 95% CI [61%, 80%], *z* = 3.86, *p* = 0.0001, *OR* = 2.51; Stage 3: *B* = 74%, 95% CI [59%, 85%], *z* = 3.01, *p* = .003, *OR* = 2.88. At the same time, like in Stage 1, expectations for the choice of others in the community was less consistent across sites. Indigenous samples considered self-reliant (vs advice-oriented) strategies as favoured by others in their community (figure 1), *B* = 70%, 95% CI [51%, 84%], *z* = 2.08, *p* = 0.034, *OR* = 2.34. Moreover, Stage 3 participants showed no conclusive expectation that advice-oriented or self-reliant strategies would be preferred by their community, overall *B* = 49%, 95% CI [36%, 62%], |*z*| = 0.13, *p* = 0.890, *OR* = 0.96.

### (b) Pre-registered analyses: cultural and individual differences in decision preferences

**Figure 3.**
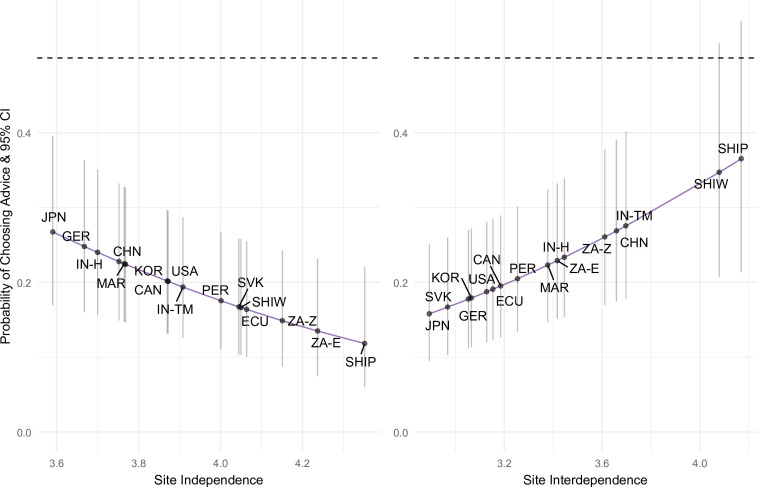
Advice‐strategy probability by site. Points (±95% CI) are GLMM site random effects (covariates in table 3). *y*-axis: probability of favouring advice; left *x*: site-level independence, right *x*: site-level interdependence. Independence associated negatively, interdependence positively, with advice use. Labels use ISO country codes (CAN, CHN, ECU, GER, JPN, KOR, MAR, PER, SVK, USA, ZAF) plus language-/community-specific tags: IN-H, IN-TM, ZA-E, ZA-Z, SHIP, SHIW.

Though we observed modal preference for self-reliant strategies in all samples, we also observed substantial cultural and individual differences in the magnitude of endorsement of advice-oriented strategies (figures 1 and 2). Samples that on average reported more independent self-construal showed a stronger preference toward self-reliant strategies (table 3 and left panel of figure 3). In contrast, samples that endorsed more interdependent self-construal and self-transcendence in reflection on social issues showed a stronger preference for advice-oriented strategies (table 3 and right panel of figure 3). Notably, the mean estimate of preference for advice-oriented (vs self-reliant) strategies was below the parity value of 0.50 in all samples (dashed line in figure 3), indicating a modal tendency towards self-reliant strategies in all samples. In other words, although more interdependent (and less independent) samples were *relatively* more likely to seek advice, even in the most interdependent samples the majority favoured self-reliant over advice-oriented strategies.

**Table 3.** Sample- and person-level factors associated with advice preferences.

	null model			sample model			sample and person model		
*predictors*	*odds*	*CI*	*p*	*odds*	*CI*	*p*	*odds*	*CI*	*p*
	*ratios*			*ratios*			*ratios*		
intercept	0.24	0.14 – 0.40	**<0.001**	2.53	0.14 – 46.71	0.533	2.34	0.08 – 65.16	0.617
*sample-level*									
independence				0.22	0.08 – 0.61	**0.004**	0.27	0.09 – 0.79	**0.017**
interdependence				2.61	1.39 – 4.90	**0.003**	2.41	1.26 – 4.60	**0.008**
need for cognition				0.87	0.35 – 2.21	0.774	0.82	0.30 – 2.24	0.695
self-transcendence				4.09	1.45 – 11.58	**0.008**	3.40	1.15 – 10.05	0.027
solution-seeking				1.14	0.32 – 4.08	0.836	1.08	0.28 – 4.20	0.910
meta-cognition				0.30	0.10 – 0.91	0.034	0.35	0.11 – 1.13	0.079
*person-level*									
independence							0.71	0.64 – 0.77	**<0.001**
interdependence							1.18	1.09 – 1.29	**<0.001**
need for cognition							0.81	0.74 – 0.89	**<0.001**
self-transcendence							1.08	1.02 – 1.14	**0.014**
solution-seeking							0.92	0.84 – 1.01	0.066
meta-cognition							1.05	0.96 – 1.15	0.266
random effects									
σ^2^	3.29			3.29			3.29		
τ_00_	0.91 _id:site_			0.91 _id:site_			0.82 _id:site_		
	0.20 _site_			0.04 _site_			0.05 _site_		
	0.35 _vignette_			0.34 _vignette_			0.38 _vignette_		
*ICC*	0.31			0.28			0.27		
*N*	6 _vignette_			6 _vignette_			6 _vignette_		
	3517 _id_			3517 _id_			3240 _id_		
	16 _site_			16 _site_			16 _site_		
observations	16 777			16 777			15 956		
marginal *R*^2^/conditional *R*^2^	—/0.308			0.028/0.303			0.040/0.304		
AICc	17 377.951			17 371.377			16 381.370		

Note. AIC = Akaike information criterion. Odds ratio>1 reflects a positive association, whereas odds ratio <1 reflects a negative association. When bolding statistically significant effects, we use a stricter α criterion (*p* < 0.02) because of large sample size and statistical power. Under such circumstances, when the null hypothesis is true, a *p*‐value = 0.04 is more likely than when the alternative hypothesis is true [37].

Whereas sample-specific variability accounted for 3% of the variance in personal preferences, 20% of the variability was accounted for by inter-individual differences, and 8% by scenario-specific variability.^4^ We now explore individual differences and cross-situational effects.

### (c) Situational differences in internal decision preferences

Is the preference for advice-discounting strategies chiefly a product of the social costs of advice-oriented strategies? Asking others whether one should help a neighbour or a friend (rather than focusing on personal gains) may have reputational costs. Thus, advice-discounting preferences may be due to the risks involved when asking others. To test this possibility, we explored situational differences in decision preferences. By design, half of our scenarios involved a choice between two equally attractive options (personal choice). The other half concerned a choice between a self-protective option and a socially considerate option of helping a neighbour or a fellow student (social choice). Comparing results across both scenario types, we observed systematic discounting for advice-oriented strategies across *both* scenario types (i.e. even when the scenario involved a personal choice between two equally attractive options, where seeking additional perspectives hardly damages one’s reputation, participants strongly favoured advice-discounting strategies). At the same time, relative preference for advice-oriented strategies was significantly lower for social choices (8.5%, 95% *CI* [4.8%, 11.6%]) compared to personal choice scenarios (24%, 95% *CI* [16.0%, 33.0%]), *OR* (personal versus social) = 3.77, s.e. = 0.87, *z* = 5.75, *p* < 0.001. Thus, while reputational costs did not explain the systematic preference for advice-discounting strategies for each scenario type, reputational considerations appear to have amplified advice-discounting preferences.

## 5. Discussion

Across twenty samples from urban and rural communities across multiple continents and including two Indigenous communities, and in the face of six everyday dilemmas devised with local collaborators, people overwhelmingly preferred self-reliant strategies. Private deliberation emerged as the modal preference in most societies, crowd wisdom the least preferred, and this hierarchy of self-reliant over advice-oriented strategies held in industrialized capitals, small towns and Amazonian rainforest villages alike. The consistency of these preferences allows a direct test of the rival accounts posed in our Introduction. Were advice-discounting merely a Western artefact born of independent self-construals, strongly interdependent cultures should have preferred friends’ or crowd advice. They did not. Instead, the self-reliance tilt appeared in all sites we tested, suggesting that motivational and cognitive biases favouring inward-looking decision routes are a human regularity that culture calibrates but does not overturn.

The apparent universality of this preference provokes a re-thinking of the independent–interdependent agency distinction that has long organized cultural psychology [12,13,38,39]. Our data show that even the most interdependent samples prefer to deliberate privately, suggesting that interdependence does not translate into appetite for explicit advice. One plausible explanation is pragmatic: in tight networks characterizing interdependent societies, requesting counsel may impose costs [20,40], signal incompetence or create new obligations [41], making inner deliberation the safer, more socially considerate option. Additionally, seeking advice could force one to disclose personal information, which may be riskier in cultures with low relational mobility [42]. By contrast, participants endorsing independent self-views appeared to choose self-reliance to affirm agency and competence. Thus, both cultural ecologies converge on the same behavioural endpoint—deciding alone—but for subtly different reasons.

Despite the modal preference for self-reliant strategies in each sample, the extent of this self-reliance tilt varied by self-construal [39,43], with persons endorsing independence showing a *relatively* stronger preference for self-reliant strategies and persons endorsing interdependence showing less advice-discounting. Additionally, persons favouring a self-transcendent perspective in their reflections of social conflicts were more open to advice. Thus, while the modal preference for self-reliance appears pancultural, dovetailing with autonomy and competence motives [44], further cultural and individual factors clearly mattered to explain the degree to which individuals embraced this preference.

Our findings also refine cultural-evolutionary theories that foreground social learning [45] and conformity [46]. Whereas these theories highlight the value of others’ knowledge, our results show that such value does not translate into seeking others’ explicit counsel. People may still observe, imitate people they trust or deploy the copy-the-majority heuristic [47], but when asked how they *prefer* to decide, they opt to keep deliberation private. Future work that pairs preference reports with behavioural traces will be needed to reveal when and how tacit advice use intrudes on this declared independence.

Several caveats deserve mention. The samples, though socio-demographically diverse (see results from the Supplementary Study in electronic supplementary material, figure S2–S6) and including two non-industrial populations, were nonetheless not nationally representative. Moreover, our dilemmas were standardized rather than lived; only field studies will determine whether expressed preferences govern real decisions or merely aspirational ones. Finally, like in most survey- and interview-based studies, it is possible that participants’ responses were influenced by social desirability of providing responses scientists wanted to hear. Yet the consistency of the present pattern across languages, religions, educational levels and modes of data collection strengthens confidence that we have identified a robust psychological regularity.

The present work challenges the intuitive equation of ‘interdependent’ with ‘advice-oriented’. Instead, it reveals a modal preference for self-reliance that cultures and individuals tune rather than invert. This finding urges scholars to move beyond the independence–interdependence dichotomy and to distinguish *how* people acquire social information (often implicitly) from *whether* they believe they need explicit advice. Recognizing this nuance sharpens theories of cultural evolution, reframes debates about agency across societies and sets a clear agenda: map the conditions under which people override their self-reliant default and actively turn to others—because when they do, human collective intelligence can finally come into its own.

## Data Availability

All preregistrations, materials, data and code are openly archived at OSF [48]. Supplementary material is available online [49].

## Footnotes

^1^
We leveraged the Geography of Philosophy Project’s existing relationships to these communities, but we do not treat them as representative of Indigenous or other non‑industrialized populations.

^2^
Item ‘I will be more happy if I make my own choices than if I listen to others’ showed close conceptual overlap with advice-discounting. Analyses without this item yield close to identical results. Furthermore, analyses on this item alone did not yield significant site-level differences (cf. Figure 3).

^3^
Personal deliberation was the most‐preferred strategy at every site (*z* ≥ 5.15, *p* < 0.001) except that Canadians and Tamil/Meitei participants equated it with gut feeling. Conversely, wisdom of crowds was generally least favoured (*z* ≥ 5.82, *p* < 0.001); Germans, South Africans and South Koreans treated it similarly to friends’ advice. Patterns replicated in Stages 2–3 (*z* ≥ 3.22, *p* ≤ 0.001); only Shiwiar, Indian and South African samples showed the noted exceptions.

^4^
Results were comparable for perceived wisdom. We performed analogous analyses for intuitive preferences: 2% were accounted for by sample, 20% by inter-individual differences and 3.6% by scenario-specific variability. Similarly, 3.2%, 25.2% and 3.5% of the variance in nomination of intuitive strategies as a wise choice were accounted for by sample, individual differences and scenario-specific variability, respectively.

## References

[1] Henrich JP. 2016 The secret of our success: how culture is driving human evolution, domesticating our species, and making us smarter. Princeton, NJ: Princeton University Press.

[2] Richerson PJ, Boyd R. 2008 Not by genes alone: how culture transformed human evolution. Chicago, IL: University of Chicago Press.

[3] Yaniv I, Kleinberger E. 2000 Advice taking in decision making: egocentric discounting and reputation formation. Organ. Behav. Hum. Decis. Process. **83**, 260–281. (10.1006/obhd.2000.2909) 11056071

[4] Bonaccio S, Dalal RS. 2006 Advice taking and decision-making: an integrative literature review, and implications for the organizational sciences. Organ. Behav. Hum. Decis. Process. **101**, 127–151. (10.1016/j.obhdp.2006.07.001)

[5] Grossmann I, Dorfman A, Oakes H. 2020 Wisdom is a social-ecological rather than person-centric phenomenon. Curr. Opin. Psychol. **32**, 66–71. (10.1016/j.copsyc.2019.07.010) 31400714

[6] Grossmann I, Kross E. 2014 Exploring ‘Solomon’s paradox’: self-distancing eliminates the self-other asymmetry in wise reasoning about close relationships in younger and older adults. Psychol. Sci. **25**, 1571–1580. (10.1177/0956797614535400) 24916084

[7] Kahneman D, Lovallo D. 1993 Timid choices and bold forecasts: a cognitive perspective on risk taking. Manag. Sci. **39**, 17–31. (10.1287/mnsc.39.1.17)

[8] Yaniv I. 2004 Receiving other people’s advice: influence and benefit. Organ. Behav. Hum. Decis. Process. **93**, 1–13. (10.1016/j.obhdp.2003.08.002)

[9] Mesoudi A, Chang L, Dall SRX, Thornton A. 2016 The evolution of individual and cultural variation in social learning. Trends Ecol. Evol. **31**, 215–225. (10.1016/j.tree.2015.12.012) 26775795

[10] Muthukrishna M, Morgan TJH, Henrich J. 2016 The when and who of social learning and conformist transmission. Evol. Hum. Behav. **37**, 10–20. (10.1016/j.evolhumbehav.2015.05.004)

[11] Morin O, Jacquet PO, Vaesen K, Acerbi A. 2021 Social information use and social information waste. Phil. Trans. R. Soc. B **376**, 20200052. (10.1098/rstb.2020.0052) 33993762 PMC8126467

[12] Markus HR, Kitayama S. 1991 Culture and the self: implications for cognition, emotion, and motivation. Psychol. Rev. **98**, 224–253. (10.1037//0033-295x.98.2.224)

[13] Heine SJ. 2008 Cultural psychology. New York, NY: Norton.

[14] Radford MHB, Mann L, Ohta Y, Nakane Y. 1991 Differences between Australian and Japanese students in reported use of decision processes. Int. J. Psychol. **26**, 35–52. (10.1080/00207599108246848)

[15] Savani K, Morris MW, Naidu NVR, Kumar S, Berlia NV. 2011 Cultural conditioning: understanding interpersonal accommodation in India and the United States in terms of the modal characteristics of interpersonal influence situations. J. Personal. Soc. Psychol. **100**, 84–102. (10.1037/a0021083) 20954782

[16] Deci EL, Ryan RM. 1985 Intrinsic motivation and self-determination in human behavior. New York, NY: Plenum.

[17] Pronin E, Lin DY, Ross L. 2002 The bias blind spot: perceptions of bias in self versus others. Personal. Soc. Psychol. Bull. **28**, 369–381. (10.1177/0146167202286008)

[18] Tversky A, Koehler DJ. 1994 Support theory: a nonextensional representation of subjective probability. Psychol. Rev. **101**, 547–567. (10.1037//0033-295x.101.4.547)

[19] Smith PB, Peterson MF, Misumi J. 1994 Event management and work team effectiveness in Japan, Britain and USA. J. Occup. Organ. Psychol. **67**, 33–43. (10.1111/j.2044-8325.1994.tb00547.x)

[20] Kim HS, Sherman DK, Taylor SE. 2008 Culture and social support. Am. Psychol. **63**, 518–526. (10.1037/0003-066x) 18793039

[21] Delhey J, Newton K, Welzel C. 2011 How general is trust in ‘most people’? Solving the radius of trust problem. Am. Sociol. Rev. **76**, 786–807. (10.1177/0003122411420817)

[22] Henrich JP, Heine SJ, Norenzayan A. 2010 The weirdest people in the world? Behav. Brain Sci. **33**, 61–83. (10.1017/s0140525x0999152x) 20550733

[23] Rad MS, Martingano AJ, Ginges J. 2018 Toward a psychology of Homo sapiens: making psychological science more representative of the human population. Proc. Natl. Acad. Sci. USA **115**, 11401–11405. (10.1073/pnas.1721165115) 30397114 PMC6233089

[24] Uskul AK *et al*. 2024 Challenges and opportunities for psychological research in the majority world. Collabra **10**, 123703. (10.1525/collabra.123703)

[25] Kahneman D, Wakker PP, Sarin R. 1997 Back to Bentham? Explorations of experienced utility. Q. J. Econ. **112**, 375–406. (10.1162/003355397555235)

[26] Kitayama S, Snibbe AC, Markus HR, Suzuki T. 2004 Is there any ‘free’ choice? Self and dissonance in two cultures. Psychol. Sci. **15**, 527–533. (10.1111/j.0956-7976.2004.00714.x) 15270997

[27] Yates JF, de Oliveira S. 2016 Culture and decision making. Organ. Behav. Hum. Decis. Process. **136**, 106–118. (10.1016/j.obhdp.2016.05.003) 32288179 PMC7126161

[28] Quintanilla P, Barrett HC, Cepek ML, Fabiano E, Machery E. 2023 Epistemologías andinas y amazónicas: conceptos indígenas de conocimiento, sabiduría y comprensión. Lima, Peru: Fondo Editorial de la PUCP.

[29] Dufner M, Gebauer JE, Sedikides C, Denissen JJ. 2019 Self-enhancement and psychological adjustment: a meta-analytic review. Personal. Soc. Psychol. Rev. **23**, 48–72. (10.1177/1088868318756467) 29534642

[30] Sedikides C, Gaertner L, Toguchi T. 2003 Pancultural self-enhancement. J. Pers. Soc. Psychol. **84**, 60–79. (10.1037/0022-3514.84.1.60) 12518971

[31] Oyserman D. 1993 The lens of personhood: viewing the self and others in a multicultural society. J. Personal. Soc. Psychol. **65**, 993–1009. (10.1037//0022-3514.65.5.993)

[32] Muthén B, Asparouhov T. 2018 Recent methods for the study of measurement invariance with many groups: alignment and random effects. Sociol. Methods Res. **47**, 637–664. (10.1177/0049124117701488)

[33] Cacioppo JT, Petty RE. 1982 The need for cognition. J. Personal. Soc. Psychol. **42**, 116–131. (10.1037//0022-3514.42.1.116)

[34] Brienza JP, Kung FYH, Santos HC, Bobocel DR, Grossmann I. 2018 Wisdom, bias, and balance: toward a process-sensitive measurement of wisdom-related cognition. J. Personal. Soc. Psychol. **115**, 1093–1126. (10.1037/pspp0000171) 28933874

[35] Grossmann I, Brienza JP, Bobocel DR. 2017 Wise deliberation sustains cooperation. Nat. Hum. Behav. **1**. (10.1038/s41562-017-0061)

[36] Rudnev M *et al*. 2024 Revised structure and validity of the situated wise reasoning scale across cultures. OSF. (10.17605/OSF.IO/84R2K)

[37] Maier M, Lakens D. 2022 Justify your alpha: a primer on two practical approaches. Adv. Methods Pract. Psychol. Sci. **5**, 251524592210803. (10.1177/25152459221080396)

[38] Liu SS, Morris MW, Talhelm T, Yang Q. 2019 Ingroup vigilance in collectivistic cultures. Proc. Natl Acad. Sci. USA **116**, 14538–14546. (10.1073/pnas.1817588116) 31249140 PMC6642384

[39] Varnum MEW, Grossmann I, Kitayama S, Nisbett RE. 2010 The origin of cultural differences in cognition: evidence for the social orientation hypothesis. Curr. Dir. Psychol. Sci. **19**, 9–13. (10.1177/0963721409359301) 20234850 PMC2838233

[40] Kim HS, Sherman DK, Ko D, Taylor SE. 2006 Pursuit of comfort and pursuit of harmony: culture, relationships, and social support seeking. Personal. Soc. Psychol. Bull. **32**, 1595–1607. (10.1177/0146167206291991) 17122173

[41] Yamagishi T. 2011 Trust: the evolutionary game of mind and society. Tokyo, Japan: Springer. (10.1007/978-4-431-53936-0)

[42] Thomson R *et al*. 2018 Relational mobility predicts social behaviors in 39 countries and is tied to historical farming and threat. Proc. Natl Acad. Sci. USA **115**, 7521–7526. (10.1073/pnas.1713191115) 29959208 PMC6055178

[43] Oyserman D, Coon HM, Kemmelmeier M. 2002 Rethinking individualism and collectivism: evaluation of theoretical assumptions and meta-analyses. Psychol. Bull. **181**, 3–72. (10.1037//0033-2909.128.1.3) 11843547

[44] Deci EL, Ryan RM. 1990 A motivational approach to self: integration in personality. In Perspectives on motivation (ed. RA Dienstbier), pp. 237–288. Lincoln, NE: University of Nebraska Press. 2130258

[45] Henrich JP, Gil-White FJ. 2001 The evolution of prestige: freely conferred deference as a mechanism for enhancing the benefits of cultural transmission. Evol. Hum. Behav. **22**, 165–196. (10.1016/s1090-5138(00)00071-4) 11384884

[46] Boyd R, Richerson PJ. 1985 Culture and the evolutionary process. Chicago, IL: University of Chicago Press.

[47] Morgan TJH, Acerbi A, van Leeuwen EJC. 2019 Copy-the-majority of instances or individuals? Two approaches to the majority and their consequences for conformist decision-making. PLoS One **14**, e0210748. (10.1371/journal.pone.0210748) 30682728 PMC6347471

[48] Grossmann I, Dorfman A, Barr K, Atari M, Porter B, Rudnev M, Machery E. 2025 Perceptions of sound judgment in decision making. OSF (10.17605/OSF.IO/7S3Z6)

[49] Grossmann I, Rudnev M, Dorfman A, Atari M, Barr K, Bencherifa A *et al*. 2025 Supplementary material from: Decision-making preferences for intuition, deliberation, friends, or crowds in independent and interdependent societies. Figshare. (10.6084/m9.figshare.c.7968280) PMC12343130 40795969

